# Cuticle structure and chemical composition of waxes in *Phaeoceros laevis* (L.) Prosk (Notothyladaceae, Anthocerotophyta)

**DOI:** 10.3389/fpls.2022.785812

**Published:** 2022-10-20

**Authors:** Tamara Machado Matos, Rafael Cruz, Denilson Fernandes Peralta, Gladys Flávia de Albuquerque Melo-de-Pinna, Déborah Yara Alves Cursino dos Santos

**Affiliations:** ^1^ Departamento de Botânica, Instituto de Biociências, Universidade de São Paulo, São Paulo, Brazil; ^2^ School of Biological Sciences, Institute of Molecular Plant Sciences, University of Edinburgh, Edinburgh, United Kingdom; ^3^ Instituto de Pesquisas Ambientais, Núcleo de Conservação da Diversidade, São Paulo, Brazil

**Keywords:** bryophytes, cuticle, gametophyte, hornwort, sporophyte, surface lipids

## Abstract

The development of a hydrophobic cuticle covering the epidermis was a crucial evolutionary novelty ensuring the establishment of land plants. However, there is little information about its structure and chemical composition, as well as its functional implications in avascular lineages such as Anthocerotophyta. The main goal of the present study was to compare the gametophyte and sporophyte cuticles of *Phaeoceros laevis*. Semithin sections were analyzed through light microscopy (LM), cuticle structure was evaluated by transmission electron microscopy (TEM) and epicuticular wax morphology was analyzed by scanning electron microscopy (SEM). Total waxes were analyzed by CG/MS, and the components were identified based on the mass spectra. A thin lipophilic layer was detected on the sporophyte surface, structured as a stratified cuticular layer, similar to the well-known structure described for vascular plants. On the other hand, the gametophyte cuticle was observed only with TEM as a thin osmiophilic layer. SEM analyses showed a film-type wax on the surface of both life phases. The wax layer was eight-fold thicker on the sporophyte (0.8 µg cm^-2^) than on gametophyte (0.1 µg cm^-2^). Possible mechanical and/or drought protection are discussed. Fatty acids, primary alcohols, and steroids were identified in both life phases, while the kauren-16-ene diterpene (3%) was detected only on the sporophyte. Although no alkanes were detected in *P. laevis*, our findings unveil great similarity of the sporophyte cuticle of this hornwort species with the general data described for vascular plants, reinforcing the conservative condition of this character and supporting the previous idea that the biosynthetic machinery involved in the synthesis of wax compounds is conserved since the ancestor of land plants.

## Introduction

Bryophyta *sensu lato* (bryophytes) comprises three groups: Marchantiophyta (liverworts), Bryophyta *sensu stricto* (mosses), and Anthocerotophyta (hornworts) (Shaw and Goffinet, 2000; Vanderpoorten and Goffinet, 2009). Recent evidence suggests that these plants form a monophyletic group, with hornworts being the sister group of Setaphyta a group formed by liverworts and mosses (Puttick et al., 2018; One thousand plant transcriptomes and the phylogenomics of green plants, 2019; Breinholt et al., 2021). Even though, the relations between bryophytes and vascular plants still need more support, especially due to the neglect of bryophytes as an object of study. These plants were the descendants of the pioneers in the conquest of the terrestrial environment, have kept much of their features, and are in a key phylogenetic position to understand the traits that enabled this transition to land (Puttick et al., 2018).

According to Shaw and Goffinet (2000), bryophytes are considered the second largest group of land plants, with approximately 20,000 species (The Plant List, 2018). The division Anthocerotophyta contains 10 genera with around 250 species (Duff et al., 2007; Chantanaorrapint, 2014), with 30 species listed for the neotropics belonging to the families Anthocerotaceae, Dendrocerotaceae, and Notothyladaceae (Gradstein et al., 2001). This last family includes four genera, with *Phaeoceros* being the largest in number of species (± 40) (Duff et al., 2007; Villarreal et al., 2010). The bryoflora in Brazil consists of 1,524 species, including 11 hornworts (Costa and Peralta, 2015), of which the genus *Phaeoceros* is the most common, represented by *Phaeoceros carolinianus* (Michx.) Prosk. and *Phaeoceros laevis* (L.) Prosk. (Brazil Flora, 2021) occurring in humid anthropogenic forest environments (Gradstein and Costa, 2003; Bordin and Yano, 2009).

Hornworts are morphologically characterized by gametophytes that grow as flat thalli with up to eight large chloroplasts, each one containing a pyrenoid. Unlike mosses and liverworts, the sporophyte of hornworts is long-lived, with indeterminate growth due to the presence of meristematic tissue at the base (Goffinet, 2000; Shaw and Goffinet, 2000; Gradstein et al., 2001; Goffinet et al., 2009; Vanderpoorten and Goffinet, 2009). Also, due to its interesting phylogenetic position, hornworts may present features of the land plant’s ancestor that may have been overlooked in case they were lost or changed in the more studied mosses and liverworts.

The plant cuticle is considered an important evolutionary novelty that contributed to the diversification and establishment of land plants (Bargel et al., 2006; Raven et al., 2014). The selection of this layer during the evolution was crucial for bryophytes establishment, helping to control permeability and evaporation of water concomitant with the land colonization process (Proctor, 1979; Clayton-Greene et al., 1985). The cuticle is an extracellular highly hydrophobic stratified layer synthesized by the epidermal cells, consisting mostly of cutin and/or cutan polymer matrix (Fernández et al., 2016). According to the prevailing model, the cutin matrix is embedded with intracuticular waxes, cell wall components, and phenolics, composing the cuticle proper layer, and covered by an outermost layer, which is in direct contact with the environment, formed by epicuticular waxes. Close to the cell wall, there is another layer formed by cutin and waxes associated with polysaccharides (Heredia, 2003; Domínguez et al., 2011; Yeats and Rose, 2013; Fernández et al., 2016; Fich et al., 2016; Philippe et al., 2020). Cuticular waxes include a variety of soluble lipids derived from fatty acids and may also contain terpenoids and flavonoids (Kunst and Samuels, 2003; Jetter et al., 2006; Kunst and Samuels, 2009; Báez-Sañudo et al., 2013). The cuticle plays a vital role in tolerance to biotic and abiotic stress factors in plants, especially in controlling nonstomatal water loss to prevent desiccation (Riederer and Schreiber, 2001; Reina-Pinto and Yephremov, 2009; Ahamad et al., 2015).

Despite recent efforts to demonstrate the properties of cuticles on the surface of both the gametophyte and sporophyte phases of bryophytes, most studies focus on Setaphyta (Proctor, 1979; Haas, 1982; Clayton-Greene et al., 1985; Neinhuis and Jetter, 1995; Koch et al., 2009; Xu et al., 2009; Budke et al., 2011; Budke et al., 2012; Buda et al., 2013; Busta et al., 2016; Matos et al., 2021a; Matos et al., 2021b) but not on hornworts.

Histochemical and transmission electron microscopy (TEM) analysis revealed the presence of a thin cuticle covering the air pores of gametophyte thalli in *Marchantia* species (Schöenherr and Ziegler, 1975). This layer was also detected through a series of studies using TEM in both life cycle stages of the Funariaceae moss species, being more stratified in the sporophyte in comparison with the gametophyte (Budke et al., 2011; Budke et al., 2012; Buda et al., 2013; Budke and Goffinet, 2016). The main wax classes identified in the gametophytes of mosses and liverworts were fatty acids, primary alcohols, alkanes, and esters (Benešová et al., 1972; Heinrichs et al., 2000; Heinrichs and Rycroft, 2001; Xu et al., 2009; Matos et al., 2021a). Additionally, aldehydes, secondary alcohols, and alkane diols have been detected in the sporophytes of some moss species (Haas, 1982; Neinhuis and Jetter, 1995; Busta et al., 2016). Recent studies found quantitative and qualitative differences in waxes of the two life cycle stages of *Funaria hygrometrica* Hedw. (Funariaceae) (Busta et al., 2016) and in three Brazilian species of Polythricaceae (Matos et al., 2021b). Regarding the morphology of epicuticular waxes, amorphous to crystalloid variations have been observed in both the gametophyte and sporophyte of moss and liverwort species (Proctor, 1979; Clayton-Greene et al., 1985; Neinhuis and Jetter, 1995; Koch et al., 2009; Heinrichs and Reiner-Drehwald, 2012; Busta et al., 2016; Matos et al., 2021a; Matos et al., 2021b).

Concerning hornworts, only one study investigated the cuticle and the morphology of the cuticular waxes on the gametophyte of *Notothylas orbicularis* (Schwein.) Sull. (Notothyladaceae) (Cook and Graham, 1998), describing the presence of a thin osmiophilic layer and a film-like wax. Besides, Pressel et al. (2014) investigated the stomatal function in *Phaeoceros carolinianus* and reported the presence of wax rodlets at the stomatal opening. Recently, Kong et al. (2020) analyzed the cuticular waxes in *Anthoceros agrestis* (Paton) Damsholt (Anthocerotaceae) and verified a thin wax coverage composed, predominantly, of fatty acids.

The present study aimed to carry out a comparative description of the anatomical, morphological and chemical aspects of the cuticle on the gametophyte and sporophyte of the cosmopolitan hornwort *Phaeoceros laevis* (Notothyladaceae), to improve knowledge about the chemistry of this group of plants and contribute to understanding the role of the cuticle in the establishment of land plants.

## Materials and methods

### Plant material collection

The plant material was collected from a greenhouse belonging to the Department of Botany of the Federal University of Santa Catarina (UFSC), Brazil, in 2019. The voucher specimen was deposited at the SP Herbarium (ac.505619). The gametophytes and sporophytes were separated, rinsed with distilled water to remove residual substrate, placed on an absorbent paper towel to remove excess water, and then immediately used to extract the cuticular wax. The samples were part of a pool of plant material for each life cycle stage, obtained from different individuals (gametophytes = ± 500 mg and sporophytes = ± 200 mg).

### Cuticle structure

For light microscopy (LM), fresh gametophyte and sporophyte fragments were fixed in paraformaldehyde (1%) and glutaraldehyde (3%) solution in sodium phosphate buffer (Karnovsky, 1964), dehydrated in serial acetone embedded in epoxy resin (Spurr, 1969). Semithin sections were made with a Leica UC6 ultramicrotome and stained with toluidine blue (O’Brien et al., 1964). For histochemical identification of lipids, semithin sections were submitted to the Sudan IV test (Bronner, 1975). The histological slides were analyzed and photographed using an IM50 image manager coupled to a Leica DMLB microscope.

For transmission electron microscopy (TEM) analyses, small fragments (± 10 mm) of the gametophyte and sporophyte were fixed in a solution containing formaldehyde (2%), glutaraldehyde (2.5%) in sodium cacodylate buffer (0.1 M), pH 7.2 (modified from Karnovsky, 1965) for 48 hours. Samples were then post-fixed in osmium tetroxide (1%) in the same buffer, dehydrated through ethanol series and propylene oxide, and embedded in EMbed 812 resin (Electron Microscopy Sciences). Semithin sections (0.3 μm thick) were made in a Leica Ultracut R ultramicrotome with a glass knife, adhered to histological slides, and stained with toluidine blue. The region of interest was selected and ultrathin sectioned (70 nm thick), adhered to copper grids (200 mesh), and contrasted with solutions of uranyl acetate (Watson, 1958) and lead citrate (Reynolds, 1963). Images were obtained using a Zeiss EM900 transmission electron microscope operating at 80 kV.

### Epicuticular wax morphology

Gametophyte and sporophyte fragments with wax and with wax removed by treatment with heated chloroform for one minute (adapted from Brune and Haas, 2011), were frozen in liquid nitrogen and then lyophilized (Lyophilizer K202, Liotop) for 24 hours (modified from Li et al., 2019). The dried fragments were fixed in stubs, coated with gold using the Balzers SCD 050 sputter coater metallizer, and observed in a scanning electron microscope (SEM) (Sigma VP and DSM 940 Electron Microscope, Carl Zeiss Inc, Oberkochen, Germany), operating at 10 kV.

### Content and chemical profile of the cuticular waxes

Gametophyte and sporophyte waxes were extracted separately by two consecutive immersions with dichloromethane of 20 seconds each (adapted from Fernandes et al., 1964). The extracts were combined, filtered, and concentrated on a rotary evaporator under reduced pressure at 50°C. The concentrated wax was transferred to previously weighed glass flasks and stored in a desiccator until reached a constant mass.

Images of the gametophyte and sporophyte were obtained with a portable digital microscope (800 x with a 2.0 mp HD camera) and the surface areas of the samples were calculated using Photoshop CS6^®^. For the sporophyte, the cylinder geometric formula was used (modified from Busta et al., 2016). The values of the total wax content of the samples correspond to an average value of two measurements obtained by gravimetry, rounded to a single decimal place, and expressed in μg cm^-2^.

Aliquots of cuticular waxes were derivatized with the addition of 50 μL of N,O-bis-(trimethylsilyl)-trifluoroacetamide (BSTFA) + 50 μL pyridine, for 1 h at 70°C in a dry bath (adapted from Chu et al., 2017). Then, they were analyzed in a gas chromatograph coupled with a mass spectrometer (GC-MS — Agilent 6850/Agilent 5975C) equipped with HP5-MS column (Agilent — 30 m × 250 μm × 0.25 μm). The initial column temperature was adjusted to 100°C for 5 min, followed by heating at 5°C min^−1^ to a final temperature of 320°C, maintained for 20 minutes. The injection volume was 1 μL with helium as carrier gas at a constant flow of 1 mL min^−1^. Injector, ion source, and quadrupole temperatures were adjusted for 300°C, 230°C, and 150°C, respectively. Mass spectra were obtained using electronic ionization (EI) at 70 eV in the full-scan acquisition mode, varying between 50 ─ 800 *m/z* and 2.66 scans s^−1^. Wax compounds (relative percentages ≥ 1%) were identified by comparison of mass spectra using NIST digital library spectra (version 2.0, 2008). Each sample was analyzed twice by GC-MS.

### Data analysis

The total relative percentage of each class and the wax homologues from both life cycle stages were tabulated and graphs were constructed using Graphpad Prism^®^ software, version 8.02.

## Results

### Comparative anatomical structure

**Figure 1:**
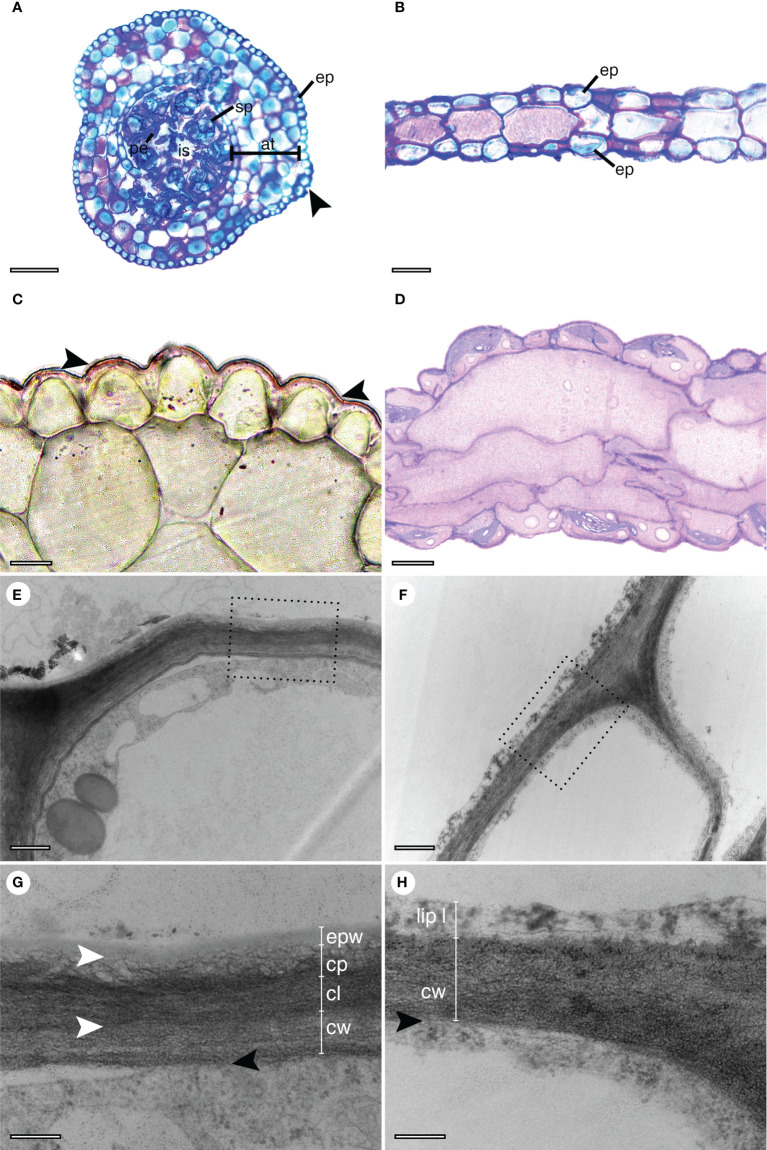
Cross-sections of *Phaeoceros laevis*. Images in the left column are sporophytes and in the right side are gametophytes. **(A–D)** Semithin sections observed under a light microscope. **(A)** Sporophyte stained with toluidine blue, with the mature capsule containing spores (sp) and pseudoelaters (pe) surround an inner space in the center. Layers of assimilative tissue (at) surround the sporogenous tissue. The entire structure is surrounded by an epidermis (ep) with a stoma (arrow). **(B)** Gametophyte stained with toluidine blue, covered by a epidermis (ep). **(C)** Thin cuticular layer (indicated by arrows) stained with Sudan IV on the sporophyte surface. **(D)** Detail of the gametophyte, stained with Sudan IV. **(E–H)** TEM. The squares in **(E, F)** are magnified in **(G, H)**, respectively. **(E)** The outer surface of an epidermal cell in the sporophyte. **(F)** The outer surface of two epidermal cells in the gametophyte. **(G)** The cell wall (cw) of the sporophyte is covered by a stratified cuticle. Cuticular layer (cl), cuticle proper (cp), and epicuticular waxes (epw) are indicated. **(H)** The cell wall (cw) of the gametophyte is covered by a lipidic layer (lip l). Bars: **(A, B)**: 100 µm, **(C, D)**: 10 µm, **(E, F)**: 0.5 µm, **(G, H)**: 0.2 µm.

Both the sporophytes and the gametophytes present a single-layered epidermis with compact cells (**Figures 1A, B**). In transverse sections, they are rounder and smaller in the sporophyte (**Figures 1A, C**) when compared with those observed in the gametophyte (**Figures 1B, D**). Stomata are only present in sporophytes (**Figure 1A**). Still in the sporophyte, four to five layers of assimilative tissue surround the sporogenous tissue with mature spores, pseudoelaters, and an inner space previously occupied by columella (**Figure 1A**). The transverse section of the gametophyte thalli shows a simpler structure with an epidermis surrounding one to three layers of bigger parenchyma cells (**Figures 1B, D**).

Under light microscopy, a thin cuticular layer was detected on the surface of the sporophyte, indicated by a positive reaction in the Sudan IV test (**Figure 1C**). However, this layer was not observed on the gametophyte (**Figure 1D**). In addition, TEM images revealed a stratified cuticular layer for the sporophyte (**Figures 1E, G**) enabling the distinction of the cuticular layer, associated with the cell wall, the proper cuticle, and epicuticular waxes. For the gametophyte, a slight osmiophilic layer external to the gametophyte was detected associated with the cell wall (**Figures 1F, H**). A thinner cell wall was observed in the sporophyte in comparison to the gametophyte (**Figures 1G, H**).

### Morphological and chemical analyses of cuticular waxes

**Figure 2:**
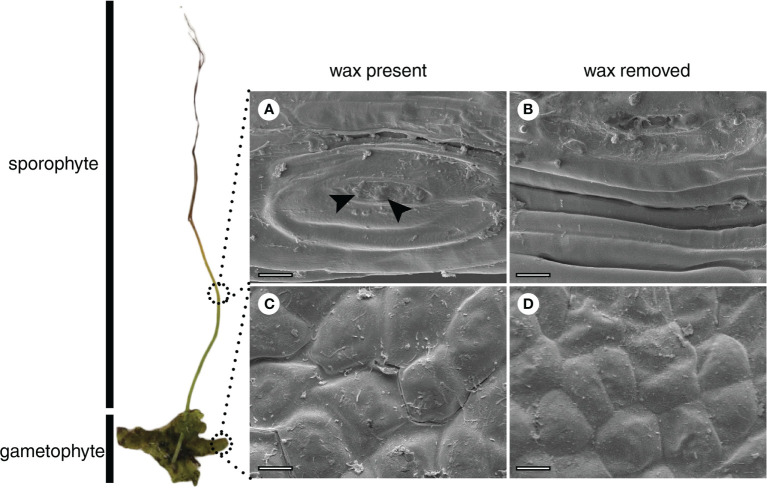
Scanning electron microscopy (SEM) of the surface of the sporophyte **(A, B)** and gametophyte **(C, D)** of *Phaeoceros laevis*. Images **(A)** and **(C)** correspond to samples with epicuticular wax, with film-like morphology. Arrows indicate possible wax granules at the sporophyte opening **(A)**. Samples **(B)** and **(D)** were treated with chloroform. Bars: 20 µm.

No variation was detected in epicuticular wax morphology. A film-like type of epicuticular wax was observed by SEM on both the sporophyte (**Figure 2A**) and gametophyte (**Figure 2C**).

**Figure 3:**
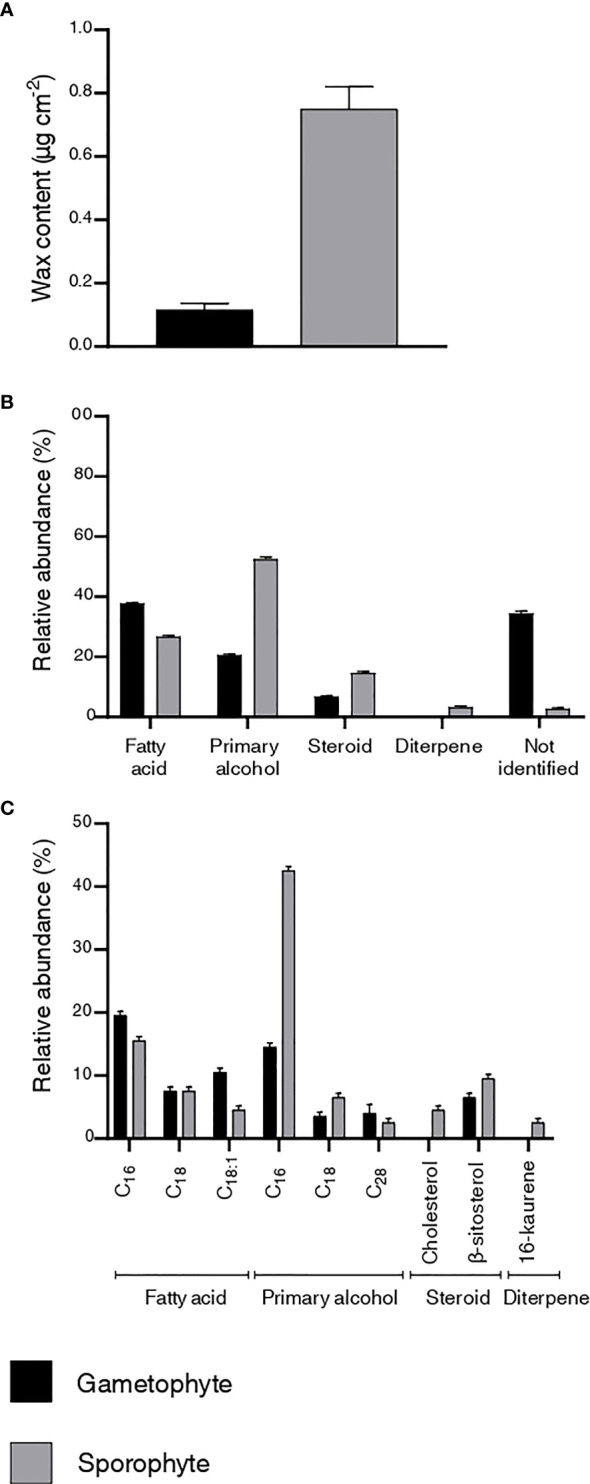
Characterization of the cuticular waxes of the gametophyte and sporophyte of *Phaeoceros laevis*. **(A)** wax content. Values for total wax content correspond to an average value of two measurements rounded to a single decimal place. **(B)** chemical profile of the wax classes. **(C)** chemical composition of wax homologues.

Quantitative and qualitative differences in the content and chemical composition of cuticular waxes were observed between *P. laevis* gametophytes and sporophytes (**Figure 3**). The wax content of the gametophyte was 0.1 µg cm^-2^, whereas an eight times thicker wax layer (0.8 µg cm^-2^) was observed on the surface of the sporophyte (**Figure 3A**). Four classes of lipids were identified in the wax chemical profiles, with fatty acids, primary alcohols, and steroids found in both life cycle stages and a diterpene detected only in the sporophyte. The main class in the gametophyte was fatty acids, with 37.7%, while primary alcohols predominated in the sporophyte, corresponding to 53% of the total wax (**Figure 3B**).

Regarding the composition of wax homologues, nine compounds were identified. Two saturated fatty acids (hexadecanoic and octadecanoic acid) and one unsaturated (cis-9-octadecenoic acid) were found in both life cycle stages, as well as the steroid β-sitosterol and the primary alcohols hexadecanol, octadecanol and octacosanol. The diterpene 16-kaurene (3%) and cholesterol (5%) were detected only in the sporophyte. The predominant homologue was hexadecenoic acid in the gametophyte, accounting for 20% of the total wax, and hexadecanol in the sporophyte, corresponding to 42% (**Figure 3C**).

## Discussion

Anatomical analyses showed a more complex structure for the sporophyte in comparison to the gametophyte, with stomata occurring only in the former. The Sudan IV test showed a thin cuticle only in the sporophyte of *P. laevis*, which was confirmed as a stratified layer by TEM. Notwithstanding, TEM observations help us identify a very thin osmiophilic layer outside the gametophyte epidermis, suggesting that the cuticle at this life stage is little lipidized. SEM analysis revealed the presence of a film-like wax layer in both life cycle stages, with an eight times thicker layer on the sporophyte surface. Fatty acids, primary alcohols, and β-sitosterol were identified in both stages, whereas 16-kaurene and cholesterol were only found in the sporophyte. Hexadecanoic fatty acid was the predominant homologue in the gametophyte and hexadecanol alcohol was the main compound in the sporophyte.

The morphological characteristics of the sporophyte and gametophyte tissues of *P. laevis* indicated by LM are very similar to those described for both life stages of *P. carolinianus* (Penjor et al., 2016). These authors indicated that these two *Phaeoceros* species are differentiated only by reproductive features, with the sporophytes more complex in cell types when compared with their gametophytes, due to specialized structures for production and dispersion of spores. In addition, once gametophytes from this genus are commonly found in humid and shady places (Penjor et al., 2016), it would be acceptable to assume that the investment in a hydrophobic structural biopolymer, such as the cuticle, is more functionally relevant to the sporophyte, which is long-lived. Besides, the presence of many layers of assimilative tissue, stomata, and a more complex cuticle in sporophytes may also be related to reduced transience, or even persistence, and its relative independence from gametophyte when compared with those of liverworts and mosses (Smith, 1938; Puttick et al., 2018; Breinholt et al., 2021).

Regarding the cuticle structure, the present study demonstrates significant differences between the sporophytes and gametophytes of *P. laevis* (**Figure 1**). A thin cuticle layer was evident with the Sudan IV test in the sporophyte of *P. laevis* (**Figure 1C**), which was confirmed as a stratified layer by TEM (**Figure 1G**). As far as we know, this is the first description of the cuticular layer for the sporophyte of hornworts. Unlike the sporophyte, no cuticle was observed on the gametophyte by LM proceedings (**Figure 1D**), but a very thin osmiophilic layer external to the gametophyte epidermis was observed by TEM (**Figure 1H**). As well in the present study, Cook & Graham (1998) described similar observations of a slight osmiophilic layer in *N. orbicularis* gametophyte using TEM. Recently, studies with leaves of vascular plant models (*Eucalyptus globulus* Labill. – Myrtaceae, *Populus* x *canescens* (Ait.) Sm. (*P. alba* L. x *P. tremula* L.) – Salicaceae, *Pyrus communis* L. var. Blanca de Aranjuez – Rosaceae) (Guzmán-Delgado et al., 2014) and with rose petals (Almonte et al., 2021), have demonstrated the presence of cell wall constituents among the cuticle layers, contradicting the traditional idea that the cuticle is free of polysaccharides and “continuous” (Brongniart, 1830; Jeffree et al., 2006). This cuticle chemical heterogeneity suggests a variation of the hydrophobic property in different regions of the same plant surface, facilitating the bidirectional permeability of water and solutes, the wettability of plant surfaces, altering mechanical resistance and, therefore, changing the interaction with microorganisms and contaminants deposited on these surfaces (Guzmán-Delgado et al., 2014; Almonte et al., 2021). The simpler cuticle detected by TEM analysis in the *P. laevis* gametophyte (**Figures 1F, H**) may be related to a lower lipidization, perhaps due to a lower wax content (**Figure 3A**), observed in the present study, and a greater presence of wall polysaccharides associated with the cuticular layer. However, this hypothesis needs a more detailed investigation of the chemical composition and functionality of the cuticle under different bryophytes surfaces.

A structure of a thin layer outermost the epidermis corresponding to the cuticle was also verified by TEM on the gametophytes of the liverwort *Marchantia* (Schöenherr and Ziegler, 1975) and of Funariaceae species (Budke et al., 2011; Buda et al., 2013). The more stratified layer in the sporophyte in comparison to that of the gametophyte found in the present study has also been found for mosses (Budke et al., 2011; Budke et al., 2012; Buda et al., 2013; Budke and Goffinet, 2016). Despite the relations between bryophytes and tracheophytes may still carry some uncertainty, based on the current phylogenetic relationships of land plants, hornworts are considered the sister group of Setaphyta, both groups separated by long phylogenetic branches (Puttick et al., 2018; One thousand plant transcriptomes and the phylogenomics of green plants, 2019; Breinholt et al., 2021). The confirmation of the monophyly of bryophytes would imply that the great structural similarity of the hornwort sporophyte cuticle with vascular plants was present in the ancestor of land plants. In addition, unlike liverworts and mosses, hornworts have a persistent sporophyte (Goffinet, 2000; Shaw and Goffinet, 2000; Gradstein et al., 2001; Goffinet et al., 2009; Vanderpoorten and Goffinet, 2009), reinforcing the idea of the crucial functional role of the cuticle in the sporophyte life stage when compared with the gametophyte. This implies the possibility of the ancestral land plant having a persistent sporophyte (supported by their complex cuticle), while the transience of this phase would be an evolutionary novelty in Setaphyta.

As observed for the gametophyte thalli of *N. orbicularis* (Cook and Graham, 1998), a film-like wax was detected in both the gametophyte and sporophyte of *P. laevis* (**Figure 2**). On the other hand, the wax rodlets observed at the stomatal opening of the *P. carolinianus* sporophyte (Pressel et al., 2014) were not identified in *P. laevis*. Although some wax granules were detected in the region of the stomatal opening before wax removal (**Figures 2A, B**), additional samples need to be analyzed to confirm this finding. According to Barthlott et al. (2017), a film-type morphology may be correlated with the predominance of alkanes, fatty acids, and primary alcohols in waxes. Our results for *P. laevis* are consistent with this suggestion, once fatty acids and primary alcohols are the main classes for the gametophyte and the sporophyte, respectively (**Figure 3B**).

Recently published data demonstrated that liverwort gametophytes have thicker layers of cuticular wax (0.1 to 4.0 μg cm^-^²) than those of mosses (0.02 to 0.6 μg cm^-^²) (Matos et al., 2021a). When comparing life cycle stages, Busta et al. (2016) observed a thicker wax layer (0.94 μg cm^-2^) in the gametophyte of *F. hygrometrica* (Funariaceae) than in the sporophyte (0.44 μg cm^-2^). Oppositely, our data show a thicker cuticular wax layer in the sporophyte (**Figure 3A**) and corroborates the results from Kong et al. (2020) that also found thinner wax coverage (<0.1 mg g^-1^) in the *A. agrestis* gametophyte. For vascular plants, the cuticle thickness is not correlated to cuticular permeability (Riederer and Schreiber, 2001; Parsons et al., 2012), once this property has been associated with the wax chemical composition (Oliveira et al., 2003), with great emphasis on alkanes. Notwithstanding, Budke and Goffinet (2016) suggested that this hypothesis of a correlation between the thickness and permeability of the cuticle in bryophytes still needs to be tested. Due to that, we speculate whether the thicker wax coverage found on the long-lived sporophyte of *P. laevis* in comparison to the habitat protected gametophyte could be related to protection against abiotic factors like mechanical and desiccation damages, compensating for the lack of alkanes. Deeper investigations on bryophytes species and its cuticle role are needed to address this hypothesis.

Fatty acids, primary alcohols, and alkanes have been identified as common classes in the waxes of gametophytes and sporophytes of different mosses (Haas, 1982; Neinhuis and Jetter, 1995; Busta et al., 2016; Matos et al., 2021a) and gametophyte of liverworts species (Benešová et al., 1972; Heinrichs and Rycroft, 2001; Matos et al., 2021a). In the case of *P. laevis*, no alkane homologs were detected in the waxes of either life cycle stage (**Figures 3B, C**). The presence of fatty acids and primary alcohol and absence of alkanes and other fatty acid derivatives are consistent with observations made by Kong et al. (2020) in *A. agrestis*. Alkanes are the most efficient wax component as a barrier against water loss (Oliveira et al., 2003) and often predominate in vascular species from environments where water is a limiting factor (Oliveira and Salatino, 2000; Oliveira et al., 2003; Kong et al., 2020). Since *P. laevis* is a species characteristic of wet environments, the lack of these components may not affect the efficiency of waxes as a barrier to nonstomatal water loss. However, this aspect also requires more in-depth research.

Regarding wax homologues, based on Kong et al. (2020) and Lee et al. (2020), the moss *Aphanoregma patens* (Hedw.) Lindb., the liverwort *Marchantia polymorpha L.* (Marchantiaceae), and the hornwort *A. agrestis* showed fatty acids (C22 to C26) and primary alcohols (C22 to C28) in cuticular waxes. However, shorter-chain fatty acids (C16, C18, C18:1) and primary alcohols (C16, C18, and C28) were observed in cuticular waxes of *P. laevis* (**Figure 3C**). Although diterpene 16-kaurene has been described as a major wax component of gametophytes in the liverwort *Anthelia julacea* (L.) Dumort. (Antheliaceae) (Huneck and Vevle, 1970), this is the first report of this compound in the sporophyte of a bryophyte. The waxes of vascular plants can also contain some classes of cyclic compounds, such as steroids, with β-sitosterol and cholesterol as the main compounds (Kunst and Samuels, 2003; Kunst and Samuels, 2009; Barthlott et al., 2017). Despite not being commonly reported in bryophyte waxes, the presence of these compounds in gametophytes and sporophytes of *P. laevis* corroborates the descriptions for other land plants.

Differences in the chemical profile of waxes between life cycle stages have also been described in bryophytes (Busta et al., 2016; Matos et al., 2021b), as observed in *P. laevis* (**Figures 3B, C**). Cholesterol and 16-kaurene were only detected in the sporophytes, corroborating the idea of Busta et al. (2016) that the expression of wax component biosynthesis pathways is defined differently in each stage of the bryophyte life cycle.

In conclusion, this is the first report of a comparative analysis of the surface structure of the sporophyte and gametophyte of a hornwort, with differences in the anatomy, content, and chemical profile of the waxes depending on the stage in the life cycle of *P. laevis*. Unreported compounds for bryophytes were identified, improving the understanding of the chemical diversity in this group of plants. In addition, our results concerning the cuticle of *P. laevis* support the conclusion of Kong et al. (2020) who suggested that part of the biosynthetic machinery involved in the cuticle formation evolved in aquatic algae. The cuticle as an important barrier to water loss evolved in the ancestor of land plants reinforced by recent molecular approaches that reveal homologies in central genes of cuticle biosynthesis between tracheophytes and bryophyte lineages, including the hornworts (Lee et al., 2020; Kong et al., 2020). That is, similarities in the cuticle structure of sporophyte *P. laevis* with vascular plants can corroborate the idea of the conserved role of this layer since the beginning of land conquest. Moreover, the presence of a complex structure of the cuticle protecting a non-transient indeterminate sporophyte is likely to be present in the ancestor of land plants, a situation reversed lately in some members of Setaphyta. Furthermore, this study paves the way for new research on the efficiency of wax layers in protecting vital structures to maintain the life cycle of bryophytes, especially hornworts, and the role of these substances in the interface between these plants and the environment.

## Data availability statement

The original contributions presented in the study are included in the article/supplementary material. Further inquiries can be directed to the corresponding author.

## Author contributions

TM, RC, GM-d-P, and DS designed the study and revised the manuscript. RC collected and DP identified the plant material and collaborated in the discussion of the data. TM prepared the extract and material for SEM analyses, and wrote the manuscript. RC and GM-d-P prepared material for histochemistry and TEM analyses. TM conducted the wax experiments and identified the wax compounds. All the authors approved the final version of the manuscript.

## Funding

Support from Coordination for the Improvement of Higher Education Personnel (CAPES, 001) to the Ph.D. scholarship of TM. . This work was supported by the Coordination for the Improvement of Higher Education Personnel (CAPES) for funding this study (funding code 001).

## Acknowledgments

We are grateful to the Department of Botany of the Universidade Federal de Santa Catarina (UFSC), Brazil, for providing the plant material, to Ph.D. student Lucas Paradizo Roma for his help in the identification of the compounds, to technicians Mourisa Ferreira and Aline Bertinatto Cruz for their assistance in the GC-MS analysis, to Irwandro Roberto Pires for his assistance in SEM analysis, and to Gisele Costa, Waldir Caldeira, Priscila Andressa Cortez and to Centro de Microscopia Eletrônica, Escola Paulista de Medicina/Universidade Federal de São Paulo (UNIFESP), Brazil, for their help processing the samples by TEM.

## Conflict of interest

The authors declare that the research was conducted in the absence of any commercial or financial relationships that could be construed as a potential conflict of interest.

## Publisher’s note

All claims expressed in this article are solely those of the authors and do not necessarily represent those of their affiliated organizations, or those of the publisher, the editors and the reviewers. Any product that may be evaluated in this article, or claim that may be made by its manufacturer, is not guaranteed or endorsed by the publisher.
